# A Multilane Tracking Algorithm Using IPDA with Intensity Feature

**DOI:** 10.3390/s21020461

**Published:** 2021-01-11

**Authors:** Behzad Akbari, Jeyan Thiyagalingam, Richard Lee, Kirubarajan Thia

**Affiliations:** 1 ECE Department, McMaster University, Hamilton, ON L8S 4L8, Canada; kiruba@mcmaster.ca; 2 Rutherford Appleton Laboratory, Scientific Computing Department, Science and Technology Facilities Council, Didcot OX11 0FA, UK; t.jeyan@stfc.ac.uk; 3 General Dynamics Land Systems—Canada, London, ON L8S 4L8, Canada; leer2@gdls.com

**Keywords:** multilane tracking, probability density function (PDF), maximum a posteriori (MAP), integrated probability data association (IPDA), curve fitting, Hough transform

## Abstract

Detection of multiple lane markings on road surfaces is an important aspect of autonomous vehicles. Although a number of approaches have been proposed to detect lanes, detecting multiple lane markings, particularly across a large number of frames and under varying lighting conditions, in a consistent manner is still a challenging problem. In this paper, we propose a novel approach for detecting multiple lanes across a large number of frames and under various lighting conditions. Instead of resorting to the conventional approach of processing each frame to detect lanes, we treat the overall problem as a multitarget tracking problem across space and time using the integrated probabilistic data association filter (IPDAF) as our basis filter. We use the intensity of the pixels as an augmented feature to correctly group multiple lane markings using the Hough transform. By representing these extracted lane markings as splines, we then identify a set of control points, which becomes a set of targets to be tracked over a period of time, and thus across a large number of frames. We evaluate our approach on two different fronts, covering both model- and machine-learning-based approaches, using two different datasets, namely the Caltech and TuSimple lane detection datasets, respectively. When tested against model-based approach, the proposed approach can offer as much as 5%, 12%, and 3% improvements on the true positive, false positive, and false positives per frame rates compared to the best alternative approach, respectively. When compared against a state-of-the-art machine learning technique, particularly against a supervised learning method, the proposed approach offers 57%, 31%, 4%, and 9× improvements on the false positive, false negative, accuracy, and frame rates. Furthemore, the proposed approach retains the explainability, or in other words, the cause of actions of the proposed approach can easily be understood or explained.

## 1. Introduction

**Figure 1:**
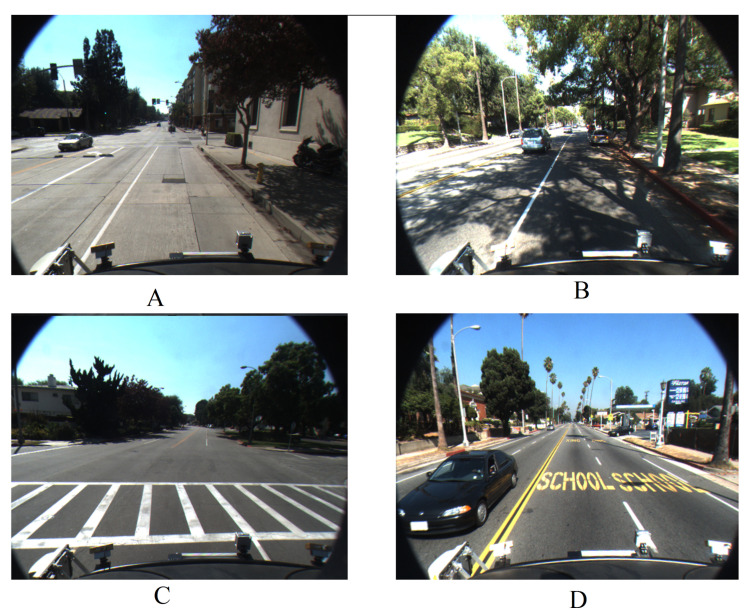
Example cases where extracting lane markings is challenging (**A**) patched road; (**B**) Shadow; (**C**) cross walk; (**D**) road markings (adopted from [18]).

Advanced driving assistance systems (ADAS) are no longer an optional or a luxurious component in modern vehicles [1,2]. Instead, they are becoming a core component, especially with the migration towards autonomous vehicles. ADAS covers a number of varying functionalities, such as lane departure warning (LDW), lane keep assist (LKA), lane change merge (LCM), adaptive cruise control (ACC), collision detection and avoidance (CD), night vision, and blind spot detection, to mention a few [1,3,4,5,6,7,8,9,10,11,12,13]. The overall functionality of the ADAS is underpinned by a machine vision component whose ability to understand the surroundings, particularly the ability to extract lane boundaries and markings in roads. With ADAS becoming a core component, it is essential that potential errors arising out of the machine vision component be as low as possible. However, correctly, consistently, and constantly extracting lane markings across a range of weather conditions is not trivial. In addition to this, varying lane marking standards, obscure lane markings, splitting and merging of lanes, and shadows of vehicles and objects exacerbate this problem even more [14,15,16,17]. We show a number of such examples in Figure 1.

The road markings can be extracted using image-based sensors like monocular or stereo vision cameras, or using LIDAR sensors. Among these, using monocular cameras is the cost-effective approach, although they lack the depth information. Stereo vision cameras can, however, provide the capability to infer the depth information and hence the ability to reconstruct three-dimensional scenarios for improved functionality, such as collision detection [4]. LIDAR sensors exploit the fact that road markings are painted using retroreflective paints. These extracted markings can then be used to extract the lane markings. However, LIDAR sensors are, similar to stereo vision cameras, far more expensive than monocular cameras. As such, seeking a trade-off between performance, reliability, and cost is an important activity in the design process. Treating cost effectiveness as the primary objective, we assume that the lane detection is performed on images obtained from a monocular camera system.

The literature on lane detection and tracking is considerably rich with a variety of techniques, covering various applications domains, including LDW, LKA, LCM, and CD. Some of these perform lane marking detection (for example, [19,20]) and track them while the rest perform only the detection (for example, [5,13,21]). In particular, we focus on techniques that solely rely on images or videos obtained from monocular vision cameras for lane marking detection followed by tracking. For instance, vision-based lane detection has been used for LDW in [5,11,12,14,22]. These approaches predominantly rely on information such as color, color cues, and edge-specific details. Color cues exploit the color contrast information between the lane markings and roads. However, the conditions have to be favorable for the differences in contrast to be realized by the lane marking algorithms. Conditions such as illumination, back lights, shadows, night lights, and weather conditions, such as rain and snow, can significantly affect the performance of color-cue-based algorithms. One approach to overcome these limitations is to use the Hough transform along with color cues [23]. However, Hough transform works well when the potential candidate lines are straight and visible enough. Although some preprocessing can improve the detection [21], consistently differentiating lane boundaries from other artifacts, such as shadows and vehicles, is a challenge.

Inverse perspective Mapping (IPM) is another approach to determine the lane boundaries in LDW systems. The central idea behind IPM is to remove the perspective distortion of lines that are parallel in real world [11,18,22]. In order to do this, images are transformed from camera view to bird’s eye view using camera parameters. During the transformation, the aspect ratios are retained so that gap or widths between lane boundaries are transformed appropriately. As such, the lane boundaries are still detectable in the transformed space. However, there are several downsides to this approach. Primarily, IPM is often used with fixed camera calibration parameters, and this may not always be optimal, owing to the surface conditions [24]. Furthermore, these transformations are computationally intensive [25], and as such, the real-time utility of these approaches needs careful implementation. Although these issues can reasonably be overcome by resorting to various techniques, such as calibration and adequate compute power systems [24,25,26], the main limitation is that the transformation is sensitive to obstacles on the road, such as vehicles, and to terrain conditions [27].

As lane markings are a pair of parallel lines, each pair should pass through a vanishing point [28]. This property can be exploited to eliminate and filter out the line segments that do not constitute lanes [29,30,31]. A number of approaches have been proposed in the literature for tracking a single lane, such as [14,17,31,32,33,34,35]. In [17], color, gradient, and line clustering information are used to improve the extraction of lane markings. In [36], an approach for lane boundary detection based on random finite sets and PHD filter is proposed as a multitarget tracking problem. In [32], a multilevel image processing and tracking framework is proposed for a monocular camera-based system. As such, it heavily relies on preprocessing of frames. Our approach also uses splines, but our tracking approach is significantly different to the one in [32]. In [33,34], techniques for personalized lane-change maneuvering are discussed. They use driver-specific behaviors, collected as part of the system, to improve the results. Although this can improve the results, such approaches are practically difficult to implement. In [35], the lane tracking is simplified by forming a midline of a single lane using B-splines. Although this approach may be useful over a short distance, conditions such as diverging lanes or missing lane markings will render the approach susceptible to bad approximations of midlines. This can easily lead to suboptimal results.

With recent advances in machine learning, particularly with supervised learning techniques such as deep learning, it is possible to engineer machine learning models to recognize lane markings. This possibility has been demonstrated in the literature [37,38,39,40,41]. In [38], a special convolutional neural network (CNN), termed spatial CNN (SCNN), was constructed for extracting the spatial correlation between objects in an image with the view of using that to establish the relative positioning of lane markings. In [39], the LaneNet consisting of two deep neural networks was constructed for lane detection. One of the networks detects lane marking edges, whereas the other network groups and clusters lane markings. The lane extraction work described in [38] relies on several integrated techniques, such as the YOLO framework [42] for object detection and convolutional patch networks (CPN) [43,44] for detecting road surfaces and regions of interest. Although these supervised techniques can offer a good result, the approach suffers from a number of issues. First, supervised learning techniques rely on labeled datasets or ground truth information. Although this appear to be trivial, these labels have to be made for each and every pixel that are to be classified as lane marking. Second, the real success of deep learning is based on the volume of data upon which the model is trained. Although the process of securing several thousands of images with labeled pixels can be automated, it is a time-consuming process. Third, training requires substantial amount of compute time. Fourth, although various supervised learning techniques can offer good accuracy rates, the explainability of the machine learning models is still an upcoming area of research, and unlike general algorithms, deep neural networks lack the rigor of explainability. This is a serious concern where lives could be at risk. Finally, the accuracy rates are never sustained across different datasets. As such, the training process is a continuous one.

This paper aims to develop a tracking technique for a multilane tracking problem based on images/videos captured from a single camera mounted in front of a vehicle. The algorithm is designed to be real-time, robust, and cost-efficient in terms of sensors. To this end, we first model each lane marking as a spline, defined by a finite set of control points. By treating these splines (and thus the control points) as targets whose motions are defined during frame transitions, we develop a multitarget tracking problem with an appropriate motion model. The multilane tracking technique proposed in this paper is a precise amalgamation of several existing real-time and robust ideas in the pipeline with the addition of certain new ideas mentioned in the contribution.

We utilize the probabilistic Hough transformation [45] to perform an initial extraction of lane markings. This is then followed by a series of algorithms prior to treating the extracted lanes as targets. The first algorithm in the pipeline performs an initial grouping of extracted line segments into different splines. This is then followed by an algorithm, which encapsulates a number of subalgorithms, to differentiate the clutter from lane boundaries in a robust manner and to manage the evolution of trajectories of splines being tracked. We then devise a multitarget tracking algorithm based on a motion model that assumes that the transitions of splines across frames are at a constant rate. The overall solution can be considered as a carefully engineered pipeline of algorithms. In doing this, we make the following key contributions:

1. We develop an algorithm, based on a maximum a posteriori (MAP) estimator [46], to group and cluster different lane segments into unknown spline groups;
2. find intensity likelihood ratio of line segments and augment this ratio as a feature in a clustering and probabilistic data association (PDA) filter [47] to distinguish lane markings from clutter; and
3. propose a new, real-time, multiple extended target tracking (targets that shape and position changed simultaneously) algorithm that works with clutter existence based on the PDA filter to distinguish and track multiple spline shape lane-lines.

The remainder of this paper is organized as follows: In Section 2, we formulate the overall problem, and discuss our approach for solving each of the subproblems. This is then followed by Section 3, in which we discuss a set of preprocessing steps on the input images prior to using our framework of methods. The aspect of clustering and estimating control points to describe the splines, and two of our key algorithms for this purpose are discussed in Section 4. We then describe the techniques to track multiple splines using the IPDA filter in Section 5. The results of our evaluations are then presented in Section 6, and we discuss conclusions in Section 7.

## 2. Problem Formulation and Our Approach

### 2.1. Problem Formulation

To facilitate the process of deriving an overall approach and suitable algorithms, we use *i* as the index for the control points i∈0,1,…,N, *j* as the lane index, and *k* as the frame index. For instance, the parameter $x_{i,j,k}$ denotes the *i*th control point for the *j*th lane on the *k*th frame. The notations used in this manuscript are given in Table 1.

With these notations in place, the overall problem of lane identification across a sequence of frames can be reformulated as follows:

1. Identification of Control Points: For a set of extracted lane markings on frame *k*, identify a set of control points that would uniquely describe each of the lane markings (assuming that lane markings do not cross each other);
2. Trajectory Management: Each candidate control point belongs to one of the lane markings, and over a period of time (across frames), these control points form distinctive trajectories if they are managed well. In order to manage these trajectories, which is a prerequisite for multitarget tracking, it is crucial to associate the control points to the trajectories they belong to; and
3. Multitarget Tracking: By using control points in each of the trajectories as pseudo measurements, formulating a multitarget tracking algorithm is essential for further extraction and identification of lane markings on the frames yet to be seen.

### 2.2. Our Approach

**Figure 2:**
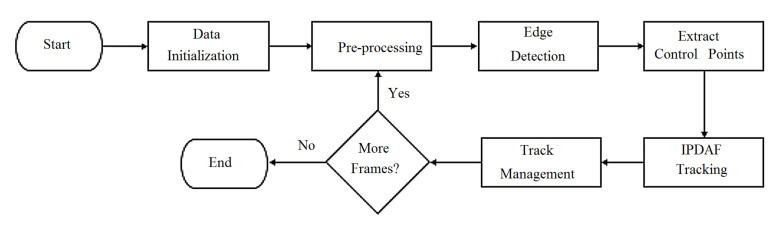
The flowchart of the proposed approach.

In addressing the overall problem outlined in Section 2.1, we decompose that into a number of subproblems, each of which handles a specific aspect of the overall lane detection problem across frames. The overall agenda is to form an automated processing pipeline, where each stage of the pipeline is underpinned by one or more algorithms. This processing pipeline is shown in Figure 2.

Each of these stages is discussed in the following sections.

## 3. Preprocessing

The key aspects of the preprocessing stage include edge detection, probabilistic Hough transform, and extraction of region of interest. We also use noise filtering before each of these stages to minimize the impact of noise amplification in the process.

### 3.1. Edge Detection

The basic edge detection in images is based on the convolution of a predetermined kernel with an image [48]. In our case, each frame forms an image. However, this basic approach for edge detection, which is a gradient finding exercise, picks up the gradients of the noise along with the lane markings. Although basic noise filtering, such as averaging or median filtering, can minimize these effects, they do not guard the edge detection from these artifacts. For this reason, we used the Canny edge detection [48], which incorporates Gaussian filtering as a precursor step to gradient calculation. More specifically, we used two 3×3 kernels, namely a Gaussian kernel *H* and an edge detection kernel *K*. For each input frame $F_{in}$, we calculated the output frame $F_{out}$ as $F_{out}=K*\left(H*F_{in}^{\prime }\right)$, where $F_{in}^{\prime }$ denotes the noise-filtered version of $F_{in}$, and * operator denotes the convolution operation. We have also prefixed the values of the *H* (by fixing the variance).

### 3.2. Probabilistic Hough Transform

Although edge detection process brings out the edges in each frame, they do not have to correspond to straight lines in the real image, particularly the lanes in the partitioned tiles. In other words, among many pixels forming different edges, the key interest is on pixels that make up straight lines—lanes. An easier implementation of this is due to [49], where an accumulator matrix captures the intersections of various straight lines in an image. This matrix is then exhaustively searched for a maximum (and other decreasing maxima) to find straight lines. As such, the original Hough transformation process is computationally intensive. In our case, we adopted the probabilistic version of Hough transform [50], where only a subset of the edge points are selected through random sampling process, particularly when updating the accumulator matrix. With this approach, we reduce the amount of computation without considering all possible measurements.

### 3.3. Extraction of Regions of Interest

Although the probabilistic Hough transform can filter out unnecessary edges and lead to straight lines, the extracted straight lines do not have to represent only the lanes. In fact, the extracted straight lines can be anything, including lanes, edges of the vehicles, lampposts, and buildings. An easier approach to filter out irrelevant components is to use the vanishing points. Each pair of lane, unlike other straight lines in a frame, should have a vanishing point.

Vanishing points can be extracted by embedding an additional process after the probabilistic Hough transform process outlined above. Sinusoids that pass through all of the maxima points in Hough space should correspond to the vanishing point in the image plane. In particular, we extract vanishing points for each partition of the image. We then use these vanishing points to eliminate irrelevant straight line segments in the image and to form regions of interest. In addition to this, the area outside the vanishing line has no information that can aid lane boundary tracking, and can be removed.

**Figure 3:**
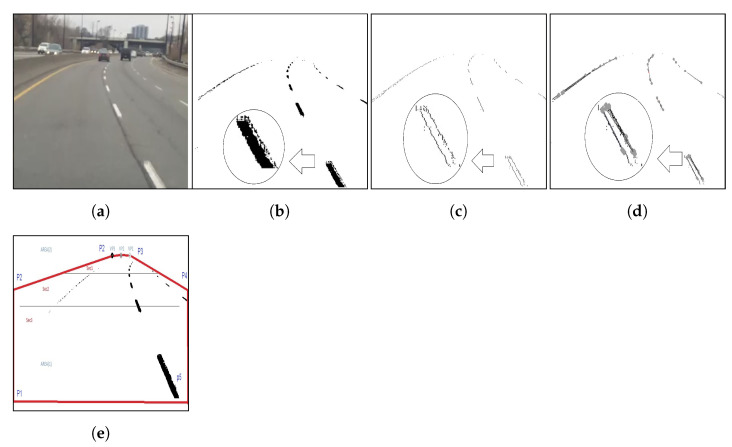
An example of preprocessing of image frames. (**a**) Raw image of the frame; (**b**) after selective segmentation; (**c**) after noise filtering and thinning; (**d**) after probabilistic Hough transform; (**e**) after extracting regions of interest.

In Figure 3, we show the outputs of different stages of the preprocessing.

## 4. Clustering and Identification of Control Points

Identification of lane-line (spline) control points starts with:

1. Partitioning frame, finding line segments and likelihood ration.
2. Predict control points and validated measurements.
3. Update final control points using MAP estimator.

### 4.1. Frame Partitioning

**Figure 4:**
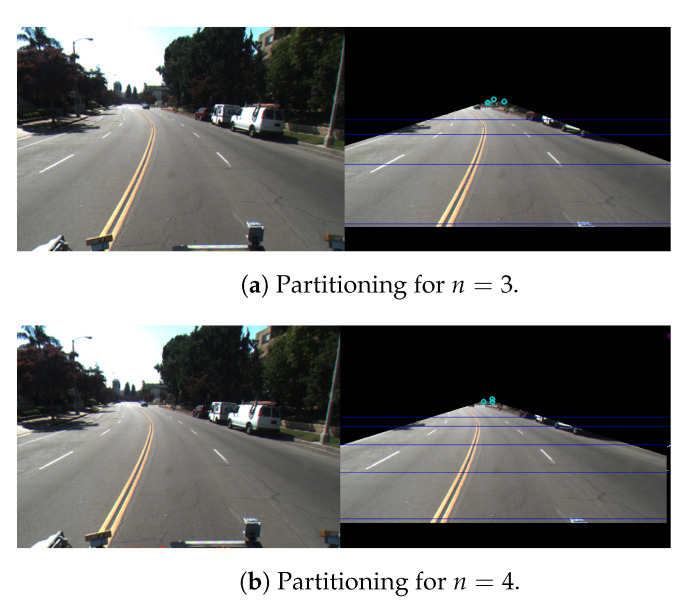
Two different examples of partitioning outputs (right) for the same input image (left). (**a**) n=3 and $h_{1}=\frac{1}{7}H$, $h_{2}=\frac{2}{7}H$ and $h_{3}=\frac{4}{7}H$; (**b**) n=4 and $h_{1}=\frac{1}{11}H$, $h_{2}=\frac{2}{11}H$, $h_{3}=\frac{3}{11}H$, and $h_{4}=\frac{5}{11}H$.

Once the preprocessing is over, the next stage of the pipeline extracts the control points. Although we intend to identify a set of control points to model the lanes as splines, the process is much simpler if the splines are small in size and straight in shape. However, the extracted lane markings are seldom straight. One approach to address this issue is to partition each frame into *n* horizontal tiles, each with an experimentally determined height, so that lanes on each partition are near straight. Figure 4 shows the same image partitioned in two different ways: for two different values of *n* (namely n=3 and n=4), and with different partition heights.

However, considering the perspective characteristics of the camera and the distance of lanes from the camera, it is beneficial to have the heights of the partitions in increasing order toward the bottom of the frame. We experimentally determined that the extracted information is maximized for n=3, such that $h_{1}=\frac{1}{7}H$, $h_{2}=\frac{2}{7}H$, and $h_{3}=\frac{4}{7}H$, where *H* is the overall height of the region of interest (ROI). We use this configuration with the values of *n* and $h_{i}$ (i=1,2,3) throughout the study conducted in this paper.

### 4.2. Intensity Likelihood Ratio of a Line Segment

For each of the partitions, we apply the probabilistic Hough transform to extract the lane markings. However, the extraction process, akin to edge in most of the detection techniques, produces a number of broken, small, noncontinuous, and irrelevant line segments. As such, one of the key challenges following the extraction process is to distinguish the lane markings from background noise and clutter. To render a more robust high-fidelity approach toward clutter and noise management, we augment the extractions with underlying intensity values. More specifically, we define the number of edge points that lie in an extended line segment *s* (s=1,…,n) as the intensity. The intensity can be extended to cover a set of line segments or a number of pseudomeasurements belonging to a curve. The intensity of an extended line segment is represented as a likelihood ratio, which we define below.

Let $p_{0}\left(f_{j}\right)$ be the probability density function (PDF) of the noise only, and $p_{1}\left(f_{j}\right)$ be the target originated line-segment detections before thresholding. Furthermore, let $D_{0}$ and $D_{1}$ be the scale parameters for false alarms and clutter, and target, respectively. These scale parameters are dependent on the minimum number of points used in the Hough transform. The noise only and target originated measurement density functions are

(1) $$p_{0}\left(f_{j}\right)=\frac{f_{j}}{D_{0}^{2}}e^{\left(\frac{-f_{j}^{2}}{2D_{0}^{2}}\right)}$$

(2) $$p_{1}\left(f_{j}\right)=\frac{f_{j}}{D_{1}^{2}}e^{\left(\frac{-f_{j}^{2}}{2D_{1}^{2}}\right)}$$

where $f_{j}\geq 0$ is the intensity of the candidate measurements *j*. Furthermore, let $\gamma =\gamma_{det}$ be the threshold to declare a detection. The probabilities of detection ($P_{D}$) and false alarm ($P_{FA}$) can be computed as follows:

(3) $$\begin{array}{ccc} P_{D} & = & \int_{\gamma }^{\infty }p_{1}\left(f_{j}\right)df_{j}\\ & = & e^{\frac{-\gamma^{2}}{2D_{1}^{2}}} \end{array}$$

(4) $$\begin{array}{ccc} P_{FA} & = & \int_{\gamma }^{\infty }p_{0}\left(f_{j}\right)df_{j}\\ & = & e^{\frac{-\gamma^{2}}{2D_{0}^{2}}} \end{array}$$

Although the probability of detection, $P_{D}$, can be increased by lowering γ, it will increases $P_{FA}$. Hence, the choice of γ cannot be arbitrary. With these, the corresponding probability density functions after thresholding become

(5) $$\begin{array}{ccc} p_{0}^{\gamma }\left(f_{j}\right) & = & \frac{1}{P_{FA}}p_{0}\left(f_{j}\right)\\ & = & \frac{f_{j}}{P_{FA}D_{0}^{2}}e^{\left(\frac{-f_{j}^{2}}{2D_{0}^{2}}\right)} \end{array}$$

(6) $$\begin{array}{ccc} p_{1}^{\gamma }\left(f_{j}\right) & = & \frac{1}{P_{D}}p_{1}\left(f_{j}\right)\\ & = & \frac{f_{j}}{P_{D}D_{1}^{2}}e^{\left(\frac{-f_{j}^{2}}{2D_{1}^{2}}\right)} \end{array}$$

where $p_{0}^{\gamma }\left(f_{j}\right)$ and $p_{1}^{\gamma }\left(f_{j}\right)$ are the probability density functions of the validated measurement $\psi_{j}$ (for j=1,…,m) that are due to noise only and originating from the target, respectively.

Considering Equations (5) and (6), the line segment intensity likelihood ratio $e_{j}$, which is the likelihood ratio of measurement $\psi_{j}$ with intensity of $f_{j}$ edge pixels originating from target rather than clutter, can be defined as

(7) $$\begin{array}{ccc} e_{j}\left(k\right) & = & \frac{p_{1}^{\gamma }\left(f_{j}\right)}{p_{0}^{\gamma }\left(f_{j}\right)}\\ & = & \frac{P_{FA}D_{0}^{2}}{P_{D}D_{1}^{2}}e^{f_{j}^{2}\left(\frac{D_{1}^{2}-D_{0}^{2}}{2D_{0}^{2}D_{1}^{2}}\right)} \end{array}$$

### 4.3. Pseudomeasurements

Pseudomeasurements $Z_{k,j}^{\tau }$ is a set of the control points for track τ and lane *j* in frame *k*. More specifically,

(8) $$\begin{array}{ccc} Z_{k,j}^{\tau } & = & \left[x_{1,j,k}^{\tau },x_{2,j,k}^{\tau },x_{3,j,k}^{\tau },x_{4,j,k}^{\tau }\right] \end{array}$$

Furthermore, let $\psi_{s}^{k}\left(j\right)$ denote the extended line segment in section *s*, at time step *k*, for lane *j*—that is, $\psi_{s}^{k}\left(j\right)$ abstracts away a number of pseudomeasurements for each (extended) line segment. Each such measurement is a two-element vector, with one capturing the pseudomeasurement $Z_{k,j}^{\tau }$ and the other one representing the intensity of the extended line segment as a likelihood ratio, $e_{j}\left(k\right)$.

### 4.4. MAP Estimator for Measurements with Intensity Feature

Although we expect the pseudomeasurements to almost model the lane-lines, in reality, a number of factors make this process as challenging. Examples include, but are not limited to, missed detection, nondeterministic nature of the preprocessing, and noisy measurements due to clutter. Therefore, it is essential to model these imperfections as part of the process.

To simplify the analysis and derivation, we assume that measurements that originate from targets at a particular sampling instant are received by the sensor only once with probability of detection $P_{D}$. The measurement equation can be written as follows:

(9) ψ(j)=x+w(j)

where j=1,…,m, w(j) is the measurement noise, and $x={\left[x_{1},x_{2}\right]}^{\prime }$ is the unknown value that we are aiming to estimate in the presence of the measurement noise.

We also assume that the measurement noise is independent and zero mean Gaussian distributed with covariance *R*. In our case, various preprocessing stages, such as thinning and Hough transform, contribute towards *R*. Thus, w(j)∼N(0,R), where

(10) $$R=\left[\begin{array}{cc} \sigma_{11}^{2} & 0\\ 0 & \sigma_{12}^{2} \end{array}\right]$$

Because of the condition of the road and perspective effect of the camera lens for values of $\sigma_{11}^{2}$ and $\sigma_{12}^{2}$, we would expect more deviation in the bottom part that is closer to the camera compared to the top. We also assume the measurements ψ to be normally distributed around *x* with covariance *R* and the prior probabilities p(x) to be normally distributed around the predicted measurement $\bar{x}$ with a covariance *Q*. Thus, ψ∼N(x,R) and $p\left(x\right)∼N\left(\bar{x},Q\right)$, where

(11) $$Q=\left[\begin{array}{cc} \sigma_{01}^{2} & 0\\ 0 & \sigma_{02}^{2} \end{array}\right]$$

Again, similar to *R*, the perspective effects of the camera influences the values of $\sigma_{01}^{2}$ and $\sigma_{02}^{2}$ to be skewed toward the bottom part of the frame. Furthermore, the covariance *Q* is often linked to the curvature κ of the road. Assuming the maximum standard curvature of highways as a constant parameter, the posterior measurement density would be

(12) $$p\left(x\Psi \right)\overset{\Delta }{=}\frac{1}{c}\left(p\left(\Psi x\right)p\left(x\right)\right)$$

Since the measurement and prior noises are assumed to be Gaussian, for a single measurement, (i.e., m=1), $\psi \left(1\right)=\psi_{1}$ can be expressed as:

(13) $$\begin{array}{ccc} p(x\psi_{1}) & \overset{\Delta }{=} & \frac{1}{c}\left(p\left(\psi 1x\right)p\left(x\right)\right)\\ & = & \frac{1}{c}N\left(\psi 1;x,R\right)N\left(x;\bar{x},Q\right)\\ & = & \frac{1}{c}N\left(x;\xi \left(\psi_{1}\right),R\right) \end{array}$$

where

$$\xi \left(\psi_{1}\right)=\frac{Q}{R+Q}\bar{x}+\frac{R}{R+Q}\psi_{1}$$

and

$$R=\frac{RQ}{R+Q}$$

For a Gaussian distribution, the mean is the optimal maximization value $\hat{x}$. Hence,

(14) $$\hat{x}=\bar{x}+\frac{R}{R+Q}\left(\psi_{1}-\bar{x}\right)$$

For m>1, the optimal maximized value can be derived using the total probability and combined residuals as follows:

(15) $$\hat{x}=\bar{x}+\frac{R}{R+Q}\sum_{j=1}^{m}\beta_{j}\left(\psi \left(j\right)-\bar{x}\right)$$

where $\beta_{j}$ is association probability, which we define as (see Appendix A for derivations). where $P_{D}$ and $P_{G}$ are probabilities of detection and gating, respectively, $m_{k}$ is the number of validated detections at time *k*, $e_{j}$ is the intensity of the extended line segments as a likelihood ratio, and $L_{j}$ is the probability density function for the correct measurement without the intensity feature, defined as

$$L_{j}=\frac{1}{P_{G}}N\left(\psi_{j},\bar{x},S\right)$$

where S=R+Q, and $\bar{x}$ is the prior information.

### 4.5. Clustering Algorithm for Finding Control Points

Ideally, each partition will have a sufficient number of full measurements $\psi^{k}\left(j\right)$ so that a spline can be fitted over those measurements. However, in reality, this is seldom the case. The associated challenges are dealt with here using an algorithm that estimates the control points based on the available set of measurements. In particular, we use the MAP estimator (MAPE) to find the optimal control points. These aspects are handled by two algorithms, Algorithms 1 and  2, which are outlined and discussed in detailed below.
**Algorithm 1** Control Points Estimator1:▹Input:κ,N,Ψ2:▹Output:A//Set of control points3:$▹\;\mathrm{Sec}\mathrm{tion}\;\mathrm{variables}\;:\;P_{i},R_{i},S_{i},\psi_{i}$4:▹κ:Vectorofcurvature5:$▹\;\psi_{i}\;:\;\mathrm{Sets}\;\mathrm{of}\;\mathrm{all}\;\mathrm{extracted}\;\mathrm{lines}\;\mathrm{in}\;\mathrm{partition}\;i\;$6:▹N:Numberofpartitions7:$▹\;P_{i}\;:\;\mathrm{Prior}\;\mathrm{noise}\;\mathrm{covariance}\;$8:$▹\;R_{i}\;:\;\mathrm{Measurement}\;\mathrm{noise}\;\mathrm{covariance}\;$9:$▹\;S_{i}\;:\;\mathrm{Set}\;\mathrm{of}\;\mathrm{partitions}\;\mathrm{indexes}\;\mathrm{eliminating}\;i\;$10:**for***i*=1; i<*N*; *i*++**do**11:    **for each** $l\in \psi_{i}$ **do**12:        **for each** $S_{i}\in \left\{1..N\right\}-\left\{i\right\}$ **do**13:           Initialize($P_{S_{i}},R_{S_{i}}$)14:           ${\bar{x}}_{S_{i},l}\leftarrow$Predict(κ,l)15:           ${\hat{x}}_{S_{i},l}\leftarrow$MAPE(${\bar{x}}_{l},\psi_{S_{i}},R_{S_{i}},P_{S_{i}}$)   //Update16:        **end for**17:        $A\leftarrow \left[\begin{array}{cc} A & {\hat{x}}_{l} \end{array}\right]$18:    **end for**19:    A=RemoveSimilarCurves(*A*)20:**end for**

Algorithm 1 handles each partition separately, but by extending the line segments into the next partition wherever needed. For a given partition *s*, it estimates the control points for each line, $x_{i,s}$, using the curvature κ. Then, the overall set of lines *L* is used to estimate the control points for that partition using the MAP estimator (see Algorithm 2). These control points are accumulated into *A* as a list. Notice that the *Predict()* function finds predicted control points for each individual line segment *l* using curvature vector κ.
**Algorithm 2** MAPE.1:$▹\;\mathrm{Inputs}:\;\bar{x},\psi ,R,P$2:$▹\;\mathrm{Output}:\;\hat{x}$3:▹Sectionvariables:P,R4:$▹\;\bar{x}\;:\;\mathrm{Priors}$5:▹ψ:Measurements6:▹P:Priornoisecovariance7:▹R:Measurementnoisecovariance8:$\psi_{validated}\leftarrow$Validated-Measurements($\bar{x},\psi ,R,P$)9:$m\leftarrow \psi_{validated}$10:**for**j=0; j<m; j++**do**11:    $r_{j}\leftarrow \psi \left(j\right)-\bar{x}$12:    $\beta_{0}\leftarrow \frac{\left(1-P_{D}P_{G}\right)}{C}$13:    $\beta_{j}\leftarrow \frac{L_{j}e_{j}}{C}$14:**end for**15:$\hat{x}=\bar{x}+\frac{R}{R+Q}\sum_{j=1}^{m}\beta_{j}r_{j}$

Algorithm 2 combines both the data association and the posteriori PDF to adjust the estimated control points. In particular, it uses the IPDA-specific target-to-track association probabilities (covering both the track existence and non-existence), $\beta_{0}$ and $\beta_{j}$ for finding the control points based on candidate control points $\bar{x}$ and measurements Ψ. More specifically,

(16) $$\begin{array}{ccc} \hat{x} & = & \underset{x}{\arg\max}\;p\left(x\Psi \right)\\ & = & \underset{x}{\arg\max}\left[p\left(\Psi x\right)p\left(x\right)\right] \end{array}$$

**Figure 5:**
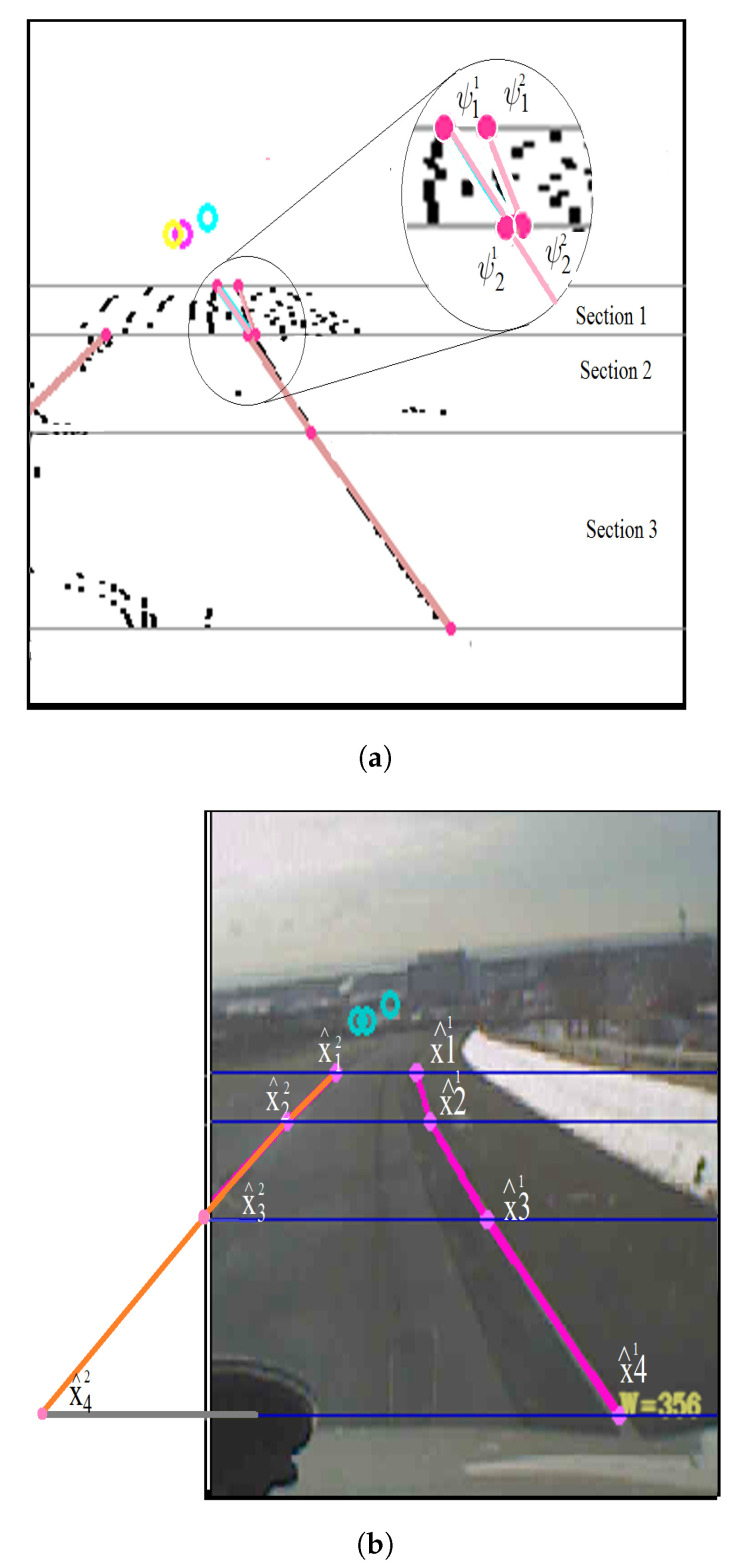
An example of control point estimation/correction and extended line segments. (**a**) Control point estimation/correction based on measurements; (**b**) grouped line segments based on the measurements.

*Validated-Measurements()* function uses normalized distance to validate the line segments belonging to each spline. We show a sample outcome of these algorithms in Figure 5. We first show two endpoint measurements $\left(\psi_{1}^{1},\psi_{2}^{1}\right)$ and $\left(\psi_{1}^{2},\psi_{2}^{2}\right)$ (Figure 5a). These points are then corrected using the above algorithms to output corrected control points $\hat{x_{1}^{1}}$ and $\hat{x_{2}^{1}}$ (Figure 5b).

## 5. Multilane Tracking Using IPDA

**Figure 6:**
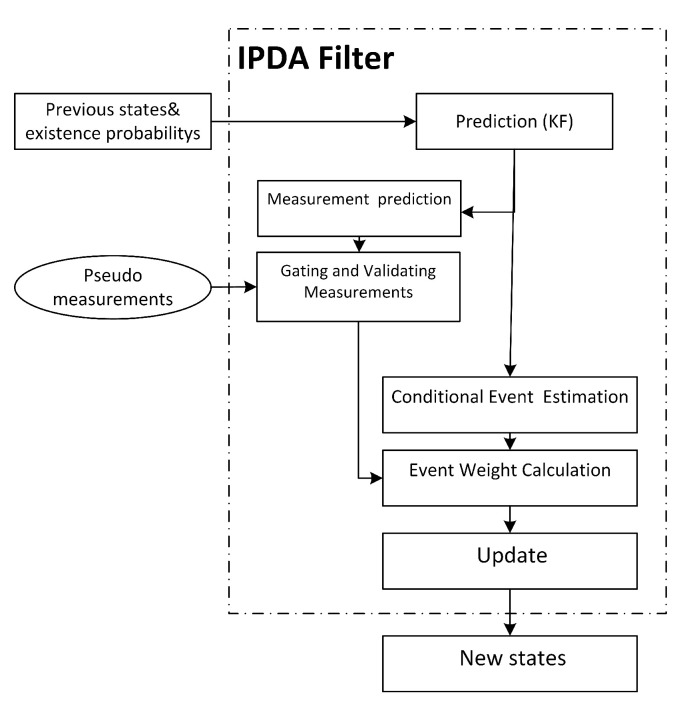
IPDAF steps.

PDA filter is similar to Kaman filter in the dynamic model and prediction, but in the update step, it uses the sum of weighted measurements to deal with clutter. We used integrated PDA (IPDA) [51] filter in the multi-target platform in a new way to combine initialization and track management for tracking multiple unknown numbers of extended targets (splines) with augmented intensity as a new feature inside association likelihood to deal with clutters, Figure 6.

### 5.1. Preliminaries

As stated above, we assume that control points are moving at constant velocity, and thus our dynamic model is a constant velocity model. With this, our state vector for track τ in frame *k* becomes

(17) $$\begin{array}{ccc} x_{k}^{\tau } & = & \left[\begin{array}{c} x_{k}^{\tau }\\ {\dot{x}}_{k}^{\tau } \end{array}\right] \end{array}$$

where

$$x_{k}^{\tau }=\left[\begin{array}{c} x_{1,k}^{\tau }\\ x_{2,k}^{\tau }\\ x_{3,k}^{\tau }\\ x_{4,k}^{\tau } \end{array}\right]\;\mathrm{and}\;{\dot{x}}_{k}^{\tau }=\left[\begin{array}{c} {\dot{x}}_{1,k}^{\tau }\\ {\dot{x}}_{2,k}^{\tau }\\ {\dot{x}}_{3,k}^{\tau }\\ {\dot{x}}_{4,k}^{\tau } \end{array}\right]$$

and the intensity feature *f* augmented (pseudo)measurement vector $Z_{k,j}^{\tau ,f}$ is

(18) $$\begin{array}{ccc} Z_{k}^{\tau ,f} & = & \left[\begin{array}{c} x_{1,k}^{\tau }\\ x_{2,k}^{\tau }\\ x_{3,k}^{\tau }\\ x_{4,k}^{\tau }\\ f_{k}^{\tau } \end{array}\right]\\ & = & \left[\begin{array}{c} x_{k}^{\tau }\\ f_{k}^{\tau } \end{array}\right] \end{array}$$

With these, the state evolution and measurement updates for frame (time) index *k* become

(19) $$x_{k}^{\tau }=Fx_{k-1}^{\tau }+G\nu_{k-1}^{\tau }+Gu_{k-1}^{\tau }$$

and

(20) $$Z_{k}^{\tau ,f}=Hx_{k}^{\tau }+\omega_{k}^{\tau }$$

where *F*, *G*, and *H* are state transition, control input, and and observation matrices, respectively, and ν and ω are measurement and process noises, respectively. In our case, we preset *F* and *G* to

(21) $$F=\left[\begin{array}{cccccccc} 1 & 0 & 0 & 0 & \Delta t & 0 & 0 & 0\\ 0 & 1 & 0 & 0 & 0 & \Delta t & 0 & 0\\ 0 & 0 & 1 & 0 & 0 & 0 & \Delta t & 0\\ 0 & 0 & 0 & 1 & 0 & 0 & 0 & \Delta t\\ 0 & 0 & 0 & 0 & 1 & 0 & 0 & 0\\ 0 & 0 & 0 & 0 & 0 & 1 & 0 & 0\\ 0 & 0 & 0 & 0 & 0 & 0 & 1 & 0\\ 0 & 0 & 0 & 0 & 0 & 0 & 0 & 1 \end{array}\right]$$

(22) $$G=\left[\begin{array}{cccc} \frac{\Delta t^{2}}{2} & 0 & 0 & 0\\ 0 & \frac{\Delta t^{2}}{2} & 0 & 0\\ 0 & 0 & \frac{\Delta t^{2}}{2} & 0\\ 0 & 0 & 0 & \frac{\Delta t^{2}}{2}\\ \Delta t & 0 & 0 & 0\\ 0 & \Delta t & 0 & 0\\ 0 & 0 & \Delta t & 0\\ 0 & 0 & 0 & \Delta t \end{array}\right]$$

To minimize the variability of the outcomes of the lane extraction process, or its dependencies on the highly variable surrounding contexts, the selection process of the control points has to be adaptive enough to account the context information. For instance, when comparing the lane extraction process on roads with varying terrain conditions opposed to highways, it is desirable if the process noise is more amenable to high variance on the spatial distribution of control points. One approach to bring the adaptiveness into this process is to introduce adaptive process and measurement noises. Furthermore, due to partitioning of frames and due to the perspective effects of the camera, control points on the bottom most partition are closer to the camera than the control points on the top-most partition of a frame. As such, despite the constant velocity model, the rates of the spatial variations of control points are different. Considering different noise variance associated with the acceleration of control points will address this issue. As such, the diagonal form of the process noise $\nu_{k}$ can be expressed as

(23) $$q=\left[\begin{array}{cccc} {\ddot{x}}_{1,k}^{\tau } & 0 & 0 & 0\\ 0 & {\ddot{x}}_{2,k}^{\tau } & 0 & 0\\ 0 & 0 & {\ddot{x}}_{3,k}^{\tau } & 0\\ 0 & 0 & 0 & {\ddot{x}}_{4,k}^{\tau } \end{array}\right]$$

where ${\ddot{x}}_{i,k}^{\tau }$ for (i=1,2,3,4) are the maximum acceleration of each control point. The process noise covariance *Q* can be expressed as

(24) $$Q=GqG^{T}$$

where

(25) $$diag_{0}\left(Q\right)={\left[\begin{array}{c} {\ddot{x}}_{1}\frac{\Delta t^{4}}{4}\\ {\ddot{x}}_{2}\frac{\Delta t^{4}}{4}\\ {\ddot{x}}_{3}\frac{\Delta t^{4}}{4}\\ {\ddot{x}}_{4}\frac{\Delta t^{4}}{4}\\ {\ddot{x}}_{1}\Delta t^{2}\\ {\ddot{x}}_{2}\Delta t^{2}\\ {\ddot{x}}_{3}\Delta t^{2}\\ {\ddot{x}}_{4}\Delta t^{2} \end{array}\right]}^{T}$$

(26) $$diag_{-4}\left(Q\right)={\left[\begin{array}{c} {\ddot{x}}_{1}\frac{\Delta t^{3}}{2}\\ {\ddot{x}}_{2}\frac{\Delta t^{3}}{2}\\ {\ddot{x}}_{3}\frac{\Delta t^{3}}{2}\\ {\ddot{x}}_{4}\frac{\Delta t^{3}}{2} \end{array}\right]}^{T}$$

and

(27) $$diag_{+4}\left(Q\right)={\left[\begin{array}{c} {\ddot{x}}_{1}\frac{\Delta t^{3}}{2}\\ {\ddot{x}}_{2}\frac{\Delta t^{3}}{2}\\ {\ddot{x}}_{3}\frac{\Delta t^{3}}{2}\\ {\ddot{x}}_{4}\frac{\Delta t^{3}}{2} \end{array}\right]}^{T}$$

where $diag_{i}\left(Q\right)$ is the *i*th subdiagonal of *Q*, with i=0 denoting the main diagonal, −i and +i denoting *i*th subdiagonals below and above the main diagonal, respectively. Similarly, the measurement noise covariance *R* is

(28) $$R=\left[\begin{array}{cccc} \sigma_{1x}^{2} & 0 & 0 & 0\\ 0 & \sigma_{2x}^{2} & 0 & 0\\ 0 & 0 & \sigma_{3x}^{2} & 0\\ 0 & 0 & 0 & \sigma_{4x}^{2} \end{array}\right]$$

### 5.2. Multilane Tracking Using IPDAF

The IPDA filter [51,52] offers an augmented information towards track maintenance apart from state estimation of tracks. Instead of assuming the existence of targets as a hard-wired probability, the IPDA filter offers a choice incorporating the track quality measure into the tracking process.

In the context of PDA algorithm, for a each validated measurement, the association probability (with each track) is calculated. To this end, the association probability $\beta_{i}^{\tau }\left(k\right)$ accounts for the probability of associating a measurement *i* to track $\tau_{k}$, feature intensity of $f_{i}^{\tau }\left(k\right)$, and the likelihood ratio of associating a line with a feature measurement $e_{i}^{\tau }\left(k\right)$. These probabilistic information are used to associate new measurements to the targets. Given a linear dynamic model, and an IPDAF based on [47], the state and measurement equations becomes

(29) $$\hat{x}\left(kk\right)=E\left[x\left(k\right)Z^{k}\right]=\sum_{i=0}^{m_{k}}{\hat{x}}_{i}\left(kk\right)\beta_{i}\left(k\right)$$

where ${\hat{x}}_{i}\left(kk\right)$ is the updated state conditioned on the event that the *i*th validated measurement being correct, and $\beta_{i}\left(k\right)$ is the probability of associating a measurement *i* with feature value of $f_{i}\left(k\right)$ to track *k*. The association probability for a set of $m_{k}$ gated or validated measurements with features $f_{i}\left(k\right)$ can be expressed as

(30) $$\beta_{i}^{\tau }\left(k\right)=P\left\{\epsilon_{i}\left(k\right)Z_{k}^{\tau },f^{\tau }\left(k\right),m_{k}\right\}$$

where $\epsilon_{i}\left(k\right)$ is the event described in Appendix A. In our case, we assume that each detected lane boundary measurement *i* has a feature of intensity $f_{i}\left(k\right)$. With reference to (A13), the feature likelihood $e_{i}\left(k\right)$ can incorporated into the PDA algorithm as follows [53]:

(31) $$\begin{array}{ccc} \beta_{i}\left(k\right) & = & P\{\epsilon_{i}\left(k\right)Z^{k},m_{k}\}\\ & = & \left\{\begin{array}{c} \frac{L_{i}\left(k\right)e_{i}\left(k\right)}{1-P_{D}P_{G}+\sum_{i=1}^{m(k)}L_{j}\left(k\right)e_{i}\left(k\right)},\forall i\neq 0\\ \frac{1-P_{D}P_{G}}{1-P_{D}P_{G}+\sum_{i=1}^{m(k)}L_{j}\left(k\right)e_{i}\left(k\right)},i=0 \end{array}\right. \end{array}$$

where i={0,1,…,m(k)}.

The overall IPDAF algorithm here embodies a traditional PDAF algorithm with special initialization and the termination steps. This is outlined in Algorithm A1 in the Appendix B. The underlying aspects of the multilane tracking follow the principles of multitarget tracking as in [47], and are discussed in the following subsections.

#### 5.2.1. Track Initialization

The track initialization process (for each track) can rely on one or two seed points. In the one-point initialization method, position can be initialized from a single observation with zero velocity vector. Due to [54],

(32) $$diag{\left(P\left(00\right)\right)}^{T}=\left[\begin{array}{c} \sigma_{1x}^{2}\\ \sigma_{2x}^{2}\\ \sigma_{3x}^{2}\\ \sigma_{4x}^{2}\\ {\left(\frac{V_{max}}{2}\right)}^{2}\\ {\left(\frac{V_{max}}{2}\right)}^{2}\\ {\left(\frac{V_{max}}{2}\right)}^{2}\\ {\left(\frac{V_{max}}{2}\right)}^{2} \end{array}\right]$$

and

(33) $$\hat{x}{\left(00\right)}^{T}=\left[\begin{array}{c} x_{1_{z}}\\ x_{2_{z}}\\ x_{3_{z}}\\ x_{4_{z}}\\ 0\\ 0\\ 0\\ 0 \end{array}\right]$$

This initialization allows the standard gating to be used during the following time step.

#### 5.2.2. Measurement Prediction

For each track, the state vector, the measurements, and the state covariance matrices are predicted ahead as in the standard Kalman filtering. i.e.,

(34) $$\begin{array}{ccc} {\hat{x}}_{kk-1} & = & F_{k-1}{\hat{x}}_{k-1k-1} \end{array}$$

(35) $$\begin{array}{ccc} P_{kk-1} & = & F_{k-1}P_{k-1k-1}F_{k-1}^{\prime }+Q_{k-1} \end{array}$$

(36) $$\begin{array}{ccc} {\hat{z}}_{kk-1} & = & H_{k}{\hat{x}}_{kk-1} \end{array}$$

(37) $$\begin{array}{ccc} S_{k} & = & H_{k}P_{kk-1}H_{k}^{\prime }+R_{k} \end{array}$$

with the assumption of Gaussian posterior for p(x) as

(38) $$p\left[x_{k-1}Z^{k-1}\right]=N\left(x_{k-1};{\hat{x}}_{k-1k-1},P_{k-1k-1}\right)$$

#### 5.2.3. Measurement Gating

For each track, a validation gate is setup around the predicted measurement to select the candidate measurements for the data association. The size of the validation gate is correlated to the innovation covariance and the measurement noise. As per (38), at most, one of the validated measurements can be assigned to the target. The measurements outside the gate are assumed to be false alarms or measurements belonging to other targets. The validation region is the elliptical shape as follows:

(39) $$V\left(k,\gamma \right)=\{z:{\left[z-{\hat{z}}_{kk-1}\right]}^{\prime }S_{k}^{-1}\left[z-{\hat{z}}_{kk-1}\right]\leq \gamma \},$$

where $n_{z}$ is the dimension of measurement vector representing the degrees of freedom, and γ is the gating threshold. Here, the gating threshold γ is a chi-squared distribution, parameterized by the probability of gating $P_{G}$, and by the degrees of freedom $n_{z}$ [46].

#### 5.2.4. Data Association

An incoming measurement at time index *k*, $z_{i}\left(k\right)$ with feature $f_{i}\left(k\right)$ is associated to track a τ, based on the association probability given by (31)

$$\begin{array}{ccc} \beta_{i}\left(k\right) & = & \left\{\begin{array}{c} \frac{L_{i}\left(k\right)e_{i}\left(k\right)}{1-P_{D}P_{G}+\sum_{i=1}^{m(k)}L_{j}\left(k\right)e_{i}\left(k\right)},\forall i\neq 0\\ \frac{1-P_{D}P_{G}}{1-P_{D}P_{G}+\sum_{i=1}^{m(k)}L_{j}\left(k\right)e_{i}\left(k\right)},i=0 \end{array}\right. \end{array}$$

Here, there are two likelihood ratios of interest: $L_{i}\left(k\right)$ and $e_{i}\left(k\right)$. The former is the likelihood ratio of the incoming measurement $z_{i}\left(k\right)$. This is defined as

(40) $$L_{i}\left(k\right)=\frac{N\left[z_{i}\left(k\right);\hat{z}\left(kk-1\right),S\left(k\right)\right]P_{D}}{\lambda }$$

where λ is the uniform density of the location of false measurements. The second parameter of interest, $e_{i}\left(k\right)$, is the likelihood ratio of measurement $z_{i}^{\tau }$ with feature $f_{i}^{\tau }$ of the track τ. This is defined as

(41) $$e_{i}\left(k\right)=\frac{p_{1}^{\tau }\left(f_{i}\right)}{p_{0}^{\tau }\left(f_{i}\right)}$$

Both of these measurements, $z_{i}\left(k\right)$ and $z_{i}^{\tau }$, are expected to originate from the target and not from the clutter.

#### 5.2.5. State Update

The state, gain, and covariance update equations of the PDAF are

(42) $$\begin{array}{ccc} \hat{x}\left(kk\right) & = & \hat{x}\left(kk-1\right)+W\left(k\right)V\left(k\right) \end{array}$$

(43) $$W(k)=P\left(kk-1\right)H{\left(k\right)}^{\prime }S{\left(k\right)}^{-1}$$

(44) $$\begin{array}{ccc} P(kk) & = & \beta_{0}\left(k\right)P\left(kk-1\right)+\\ & & \left(1-\beta_{0}\left(k\right)\right)P^{c}\left(kk\right)+\tilde{P}\left(k\right) \end{array}$$

where W(k) is the Kalman gain, and V(k) is the combined innovation defined by

(45) $$V\left(k\right)=\sum_{i=1}^{m(k)}\beta_{i}\left(k\right)V_{i}\left(k\right)$$

where $m_{k}$ is the number of measurements inside the gating region, and $\beta_{0}\left(k\right)$ is the probability of all measurements being incorrect at time index *k*. With no information on which of the $m_{k}$ measurements being correct or incorrect, the correct updated covariance can be expressed as

(46) $$\begin{array}{ccc} P^{c}\left(kk\right) & = & P\left(kk-1\right)-W\left(k\right)S\left(k\right)W{\left(k\right)}^{\prime } \end{array}$$

(47) $$\begin{array}{ccc} \tilde{P}\left(k\right) & = & W\left(k\right)\{\sum_{i=1}^{m(k)}\beta_{i}\left(k\right)\nu_{i}\left(k\right)\nu_{i}{\left(k\right)}^{\prime } \end{array}$$

(48) $$\begin{array}{ccc} & & -\nu \left(k\right)\nu {\left(k\right)}^{\prime }\}W{\left(k\right)}^{\prime } \end{array}$$

#### 5.2.6. Track Management

During the course of tracking, several tracks are maintained in parallel, and their states are continuously updated upon receiving measurements. A track can be in three different states: tentative, confirmed and terminated. During the initialization phase, every unassociated measurements will form a tentative track. However, upon following detections or measurements, and gating operations, the tracks will begin to form and their status will be updated as confirmed. However, if no further measurement to track associations are possible, a possibility when no detections are observed from the target responsible for the track, corresponding track is terminated. For the case where $P_{D}<1$, it is essential to check the quality of measurement to track association before engaging in track status update. One of the approaches for assessing the quality of measurement to track association is the goodness of fitting. The goodness of fitting, often represented by the log-likelihood function of the track, can be expressed as a recursive function as follows [46]:

(49) $$\lambda \left(k\right)=\lambda \left(k-1\right)+V{\left(k\right)}^{\prime }S{\left(k\right)}^{-1}V\left(k\right)$$

where V(k) is the innovation matrix, and S(k) is the covariance of the innovation matrix V(k). The last term in (49) has a chi-squared density with $n_{z}$ degrees of freedom, where $n_{z}$ is the dimensionality of the measurement vector. As the innovations are independent, the log-likelihood function at time *k* is a chi-squared distributed with $kn_{z}$ degrees of freedom. This is actually a measure of the goodness of fit to the assumed target model. Thus, the test function for keeping (or terminating) a track can be expressed as

(50) $$\begin{array}{ccc} \lambda^{k} & \leq & \lambda_{max}^{k} \end{array}$$

(51) $$\begin{array}{ccc} \lambda_{max}^{k} & = & X_{kn_{z}}^{2}\left(1-\alpha \right) \end{array}$$

where the tail probability α is the probability that a true track will be rejected. In our case, this threshold is around 0.01. However, the actual state transitions are performed through more rigorous checks. In our case, we maintain a number of (hidden) measurement and association counters for each track. These counters are used to assess the activeness of the measurement-track association.

## 6. Experiments and Evaluations

The proposed algorithm has been evaluated against two different baselines: model- and machine-learning-based implementations. The proposed algorithm was implemented using the OpenCV library in C++. The algorithms were tested on a system with Intel i7 CPU, clocked at 2.9 GHz with 16 GB RAM.

### 6.1. Evaluation against Model-Based Approaches

The proposed approach was benchmarked against two model-based methods, namely [18,55] using the Caltech Lane dataset [18]. The dataset has four video clips taken at different times of a day around the urban area of Pasadena in California. Each of these video clips has a resolution of 640×480, and covers various lighting and illumination conditions, writings along with lane markings (Clip#1), sun glint and different pavement types (Clip#2), shadows and crosswalks (Clip#3), and congested settings (Clip#4). As such, they are reasonably representative enough of various challenging conditions for tracking lane markings. In total, 1224 frames and 4399 lane boundaries were processed. The details of these video clips are given in Table 2. Notice that ROI parameters needs to be set in our method similar to [18,55]. Our algorithm does not need camera parameters, but since  [18,55] use IPM mapping, they need those parameters to be set.

During evaluation, we computed the true and false positive rates (TPR and FPR, respectively), where the TPR is the ratio of the number of detected lane boundaries to the number of target lane boundaries and the FPR is the ratio of the number of false positives to the number of target lane boundaries. The frames were processed at the rate of seven frames per second similar to that of other methods in the literature [18,55]. In addition to TPR and FPR metrics, we also included another metric, false positives per frame (FP/frame or FPF), which is an average of false positives across all frames. One could equally consider the true positives per frame rate as well. We used the TPR, FPR and FP/Frame as the metrics of evaluation on for model-based approaches. The results of the evaluation are shown in Table 3.

Noting that higher TPR, lower FPR, and lower FP/frame values are desirable, we highlight the best (boldface) and second best results (underlined) in the results outlined in Table 3. A number of observations can be made here:

- The TPR performance of the proposed algorithm is consistently higher than those of the other two algorithms throughout all video clips. The performance of the proposed algorithm over Clip#3 is significantly higher than that of the method in [55];
- The FPR performance of the proposed algorithm is always better than that of the method in [18];
- The FPR performance of the proposed algorithm performs better than that of the method in [55] except for Clip#4. One potential reason for this sub-optimal performance for this clip can be attributed to the difficulties in association in congested settings; and
- The FP/Frame performance is mixed across the cases.

In overall, the proposed approach outperforms the other two methods across all cases. The overall performance differences are 5%, 2%, and 2% for TPR, FPR, and FPF cases when compared against the second best version, namely the method in [55]. To ensure the operation, we manually analyzed the datasets to label all visible and invisible lane markings as target lane markings.

### 6.2. Evaluation against Deep Learning-Based Approaches

In this setting, we evaluated the proposed method against the spatially convolutional neural network (SCNN) method [38] using the TuSimple data-set [56]. The TuSimple dataset has about 7000 one-second-long video clips, each with 20 frames. The ground-truth information is available only for the last frame (frame 20) of each clip, including height and width values corresponding to lanes. Although the TuSimple dataset includes a number of road conditions, such as, straight lanes, curvy lanes, splitting and merging lanes, and shadows, we used only the straight and curvy lane conditions. Notice that like all fully supervised models the algorithm in [38] uses the trained parameters coming from training process, but we only use parameters for ROI in preprocessing step.

In addition to using TP and FN, we also use the accuracy and inference time as additional metrics of our evaluation. The overall results are shown in Table 4.

From these results, it can be seen that the proposed method outperforms the baseline method across all metrics. More specifically, the proposed method offers additional 4% improvement in accuracy along with ninefold speedup.

## 7. Conclusions

In this paper, we proposed a novel approach for multilane detection. By using the intensity feature in conjunction with the probabilistic Hough transform, we first formulated an algorithm for detecting and correctly grouping multiple lane markings. By using these lane marking as splines, we then identify a set of control points, which then get tracked over time across frames. Our evaluations, covering both model-based and machine-learning-based approaches show that it can easily outperform the model-based approaches while being suboptimal compared to the deep-learning-based approaches. However, there are a number of issues remain to be addressed. For instance, machine learning models do not provide any explanation or reasons for their decisions compared to filter-based approaches like one presented here. As such, the proposed approach embodies sufficient explainability for its actions. Further investigations are needed to establish how the performance of the proposed approach can be improved.

## Figures and Tables

**Table 1:** Symbols adopted in this manuscript.

Symbol	Description
$x_{i,k,j}$	Single control point in lane-line(spline) *j* on frame *k*
$Z_{k,j}$	Pseudomeasurements (control points) for a spline *j* on frame *k*
*R*	Covariance of the measurement noise
*Q*	Covariance of the process noise
τ	Track index
*F*	State transition matrix
*H*	Measurement matrix
$P_{G}$	Gating probability
$e_{k,j}$	Event on the *j*th spline at the *k*th frame
$\Psi_{k,j}$	Line segment measurements for lane-line *j*

**Table 2:** Caltech dataset used in the evaluation.

Clip ID	Clip Name	No. of Frames	No. of Lane Boundaries
1	cordoval1	250	975
2	cordoval2	406	1131
3	washington1	336	1329
4	washington2	232	964

**Table 3:** Comparison of our approach with other lane detection algorithms.

	Method in [18]			Method in [55]			Proposed		
	TPR	FPR	FP/Frame	TPR	FPR	FPF/Frame	TPR	FPR	FP/Frame
Clip#1	0.823	0.099	0.384	0.892	0.125	0.488	0.899	0.093	0.405
Clip#2	0.839	0.224	0.672	0.865	0.209	0.628	0.870	0.166	0.535
Clip#3	0.934	0.148	0.542	0.850	0.111	0.408	0.937	0.107	0.455
Clip#4	0.890	0.102	0.418	0.898	0.063	0.259	0.974	0.099	0.424
Overall	0.871	0.148	0.529	0.874	0.131	0.469	**0.920**	**0.116**	**0.454**

**Table 4:** Proposed approach compared with one of the deep-learning-based models (SCNN).

	SCNN [38]				Proposed			
	FPR	FNR	Accuracy	Frame/s	FPR	FNR	Accuracy	Frame/s
Straight Highway#1	0.166	0.250	0.855	0.71	0.160	0.250	0.880	6.5
Curvy Highway#2	0.479	0.416	0.825	0.72	0.166	0.250	0.861	6.5
Overall	0.375	0.361	0.836	0.71	**0.160**	**0.250**	**0.869**	**6.5**

## Appendix A. Association Probability for Lane Measurements

Assuming there are *m* detections at time *k*, when finding the association probability $\beta_{j}$ between a measurement and a target (lane *j*), the overall event ϵ(j) is comprised of two mutually exclusive events: either ϵ(j) is such that the measurement ψ(j) is from the target, for j=1,…,m, or all measurements are false.

Here, the association set $\left\{\beta_{j}\right\}$ is defined as the probability of the events $\left\{\epsilon_{j}\right\}$ given all the measurements Ψ, $\beta_{j}=p\left\{\epsilon_{j}\Psi \right\}$. In this appendix, it is shown how $\beta_{j}$ is related to the feature likelihood ratio of the measurement, $e_{j}$.

Let the set of validated measurements at time *k* be denoted as $\Psi_{k}=\left\{\psi_{k}\left(j\right)\right\}$, for $i=1,\ldots ,m_{k}$. In general, the following criteria must be satisfied:

(A1) $${\left(\psi -\bar{x}\right)}^{\prime }{\left(R+Q\right)}^{-1}\left(\psi -\bar{x}\right)\leq \gamma$$

where the γ is chi-squared distributed with $n_{z}$ degrees of freedom. The association probability of $\beta_{j}$ for a set of gated measurements can then be stated as $\beta_{j}=p\left\{\epsilon_{j}\Psi ,m_{k}\right\}$. Using Bayes’s rule, this can be expressed as

(A2) $$\beta_{j}=\frac{1}{c_{1}}p\left\{\Psi \epsilon_{j},m_{k}\right\}p\left\{\epsilon_{j}m_{k}\right\}$$

Assuming that the false measurements are uniformly distributed within the validation region, and the correct measurement location is assumed to be Gaussian with mean $\bar{x}$ and covariance of S=R+Q, the probability density function for the correct measurement without the intensity feature is

(A3) $$L_{j}=p\left\{\Psi \epsilon_{j},m_{k}\right\}=P_{G}^{-1}N\left(\psi_{j}\bar{x},S\right)$$

where $P_{G}$ is the gating probability. Since the intensities are independent of location within the validation region, the probability density function of a single correct measurement, including the intensity feature, can be expressed as the product of the intensity likelihood ratio with $L_{j}$ as follows:

(A4) $$p\left\{\psi \epsilon_{j},m_{k}\right\}=P_{1}^{\tau }\left(f_{j}\right)L_{j}$$

and for incorrect measurements

(A5) $$p\left\{\psi \epsilon_{j},m_{k}\right\}=P_{0}^{\tau }\left(f_{j}\right)V^{-1}$$

where *V* is the volume of the validation region so for the all measurements, the probability density function is

(A6) $$p\left[\Psi \epsilon_{j},m_{k}\right]=P_{1}^{\tau }\left(f_{j}\right)\left[\prod_{j=1}^{m}p_{0}^{\tau }\left(f_{j}\right)\right]V^{-m+1}e_{j}$$

for $j=1,\ldots ,m_{k}$ and

(A7) $$p\left[\Psi \epsilon_{j},m_{k}\right]=V^{-m}\left[\prod_{j=1}^{m}p_{0}^{\tau }\left(f_{j}\right)\right]$$

for j=0.

Using a nonparametric model considering

(A8) $$p\left[\epsilon_{j}m_{k},x\right]=P_{D}P_{G}m^{-1}$$

for $j=1,\ldots ,m_{k}$ and

(A9) $$p\left[\epsilon_{j}m_{k},x\right]=1-P_{D}P_{G}$$

for j=0, the association probability $\beta_{j}$ can be expressed as

(A10) $$\beta_{j}=P_{1}^{\tau }\left(f_{j}\right)\left[\prod_{j=1}^{m}p_{0}^{\tau }\left(f_{j}\right)\right]V^{-m+1}e_{j}P_{D}P_{G}m_{k}^{-1}$$

for $j=1,\ldots ,m_{k}$ and

(A11) $$\beta_{0}=V^{-m}\left[\prod_{j=1}^{m}p_{0}^{\tau }\left(f_{j}\right)\right]\left(1-P_{D}P_{G}\right)$$

for j=0.

Since the set of events $\left\{\epsilon_{j}\right\}$ are mutually exclusive and exhaustive,

(A12) $$\beta_{0}+\sum_{j=1}^{m_{k}}\beta_{j}=1$$

With some simplification, the overall results can be stated as

(A13) $$\begin{array}{ccc} \beta_{j} & = & p\{\epsilon_{j}\psi_{j},m_{k}\}\\ & = & \left\{\begin{array}{c} \frac{L_{i}e_{i}}{1-P_{D}P_{G}+\sum_{j=1}^{m_{k}}L_{j}e_{j}},\wedge j\neq 0\\ \frac{1-P_{D}P_{G}}{1-P_{D}P_{G}+\sum_{j=1}^{m_{k}}L_{j}e_{j}}\wedge j=0 \end{array}\right. \end{array}$$

## Appendix B. IPDAF-Based Lane Tracking Algorithm (Detailed)

The overall IPDAF algorithm using the PDAF algorithm with special initialization and the termination steps is outlined in Algorithm A1 below.

The algorithm assumes a composite data-type that is capable of capturing the evolution of the states over a period of time. As such, we use the dot notation to extract these properties. For instance, in our algorithm we use Γ as a variable that has all the information of all the tracks being considered in the problem. Various properties, such as *track type* and *track ID* (a unique identifier for each track), are extracted using the stated dot notation. Furthermore, the algorithm assumes the presence of the following auxiliary functions:

- getTrackType(): which returns the type of the track *i*, as temporary, retired or active;
- getTrackID(): which returns the unique identifier for the track;
- mergeTracks(): which fuses the supplied set of tracks; and
- cleanTracks(): removes dead tracks from the list.

**Algorithm A1** IPDATracker.
1: ▹Inputs:Γ,Ψ 2: ▹Output:Γ(Updated) 3: $▹\;\mathrm{Sec}\mathrm{tion}\;\mathrm{Variables}\;:\;\Lambda ,\alpha ,\alpha_{T},\Omega$ 4: $▹\;\bar{x}\;:\;\mathrm{Priors}$ 5: ▹Ψ:Measurements 6: ▹Γ:Compositedatastructurefortracks 7: ▹Λ:AcopyofΓ 8: ▹Ω:Asetcontainingassociatedtracks 9: ▹α:Atemporaryvariable 10: $▹\;\alpha_{T}\;:\;A\;\mathrm{temporary}\;\mathrm{set}\;\mathrm{of}\;\mathrm{tracks}$ 11: Λ=Γ.Tracks 12: Ω←∅ 13: **if** Λ.size()>0 **then** 14: **for** i=0; i<size(Γ); i++ **do** 15: ${\hat{x}}_{kk-1}^{i}\leftarrow$ Equation (34) 16: $P_{kk-1}^{i}\leftarrow$ Equation (35) 17: $W_{k}^{i}\leftarrow$ Equation (43) 18: ${\hat{z}}_{kk-1}\leftarrow$ Equation (36) 19: $S_{k}\leftarrow$ Equation (37) 20: V(k,γ)← Equation (39) 21: **if** V.size()>0 **then** 22: **for** j=0 **to** V.size() **do** 23: $L_{k}^{i}\leftarrow$ Equation (40) 24: $e_{k}^{i}\leftarrow$ Equation (41) 25: **end for** 26: $\beta_{k}^{i}\leftarrow$ Equation (31) 27: ${\hat{x}}_{kk}^{i}\leftarrow$ Equation (42) 28: $P_{kk}^{i}\leftarrow$ Equation (44) 29: **else** 30: $\Lambda^{i}.x_{k}\leftarrow {\hat{x}}_{k}^{i}$ 31: $\Lambda^{i}.P_{k}\leftarrow P_{k}^{i}$ 32: Ω←Ω∪0 33: $\alpha \leftarrow \mathrm{getTrackType}(\Omega ,\Lambda^{i})$ 34: $\Lambda^{i}.ID\leftarrow \mathrm{getTrackID}\left(\alpha ,\Lambda^{i}\right)$ 35: $\Lambda^{i}.type\leftarrow \alpha$ 36: **end if** 37: $\Lambda^{i}.x_{k}\leftarrow {\hat{x}}_{kk}^{i}$ 38: $\Lambda^{i}.P_{k}\leftarrow P_{kk}^{i}$ 39: Ω←Ω∪1 40: $\alpha \leftarrow \mathrm{getTrackType}(\Omega ,\Lambda^{i})$ 41: $\Lambda^{i}.ID\leftarrow \mathrm{getTrackID}\left(\alpha ,\Lambda^{i}\right)$ 42: $\Lambda^{i}.type\leftarrow \alpha$ 43: **end for** 44: Γ.NonAsscociated←Ψ−Ω 45: $\alpha_{T}\leftarrow \mathrm{mergeTracks}\left(\Lambda \right)$ 46: $\Gamma .Tracks\leftarrow \mathrm{cleanTracks}(\alpha_{T})$ 47: **else** 48: Γ.NonAsscociated←Ψ 49: **end if** 50: **return** Γ
